# Post-buckling behaviors of thin-film soft-substrate bilayers with finite-thickness substrate

**DOI:** 10.1038/s41598-022-08136-w

**Published:** 2022-03-08

**Authors:** Meng Li, Bohua Sun

**Affiliations:** School of Civil Engineering and Institute of Mechanics and Technology, Xi’an University of Architecture and Technology, Xi’an, 710055 China

**Keywords:** Physics, Applied physics

## Abstract

Surface buckling behaviors of thin-film soft-substrate bilayers have important research value. Recent research has focused on bilayers with infinite-thickness substrates. However, bilayers with finite-thickness substrates widely exist. To study this problem more comprehensively, we extended the stability theory of a beam on an elastic foundation to bilayers and then established a finite element method of bilayers using the neo-Hookean hyperelastic constitutive model. A self-contact analysis method was coupled to the finite element method so that the further buckling evolution of the film surface after folding could be simulated. Based on our analysis of various modulus ratios and thickness ratios, the evolution of the buckling path was significantly influenced by the thickness ratio. Without considering the situation of a prestressed substrate, four new buckling paths were found. Thus, we extended the single buckling path (under infinite thickness substrate) to five types. Our study also found that for path four, the substrate with a certain thickness exhibited a special final stable surface morphology. That is, regardless of the friction, a new periodic morphology after film folding appeared due to the contact slip of the film surface. Finally, further analysis showed that these five buckling paths were all dependent on different modulus ratios and thickness ratios.

## Introduction

The film surfaces of thin-film soft-substrate bilayers buckle under in-plane loading. For example, the wrinkling of human skin tissue^1^, the surface folds of a macromolecule^2–4^, the surface morphologies of air-dried plants^5,6^, the development morphologies of cerebral sulci^7,8^, the capillary wrinkling of floating elastic thin film^9^, and the wrinkling of mountain ranges^10^ are all related to buckling instability. Therefore, it is important to study the buckling problem of these structures. Most of the previous studies focused on cases in which the substrate thickness was much larger than the film^11–18^, that is, the function of the substrate can be equivalent to a semi-infinite elastic subgrade^15^. Cheng and Xu .^16^, Pocivavsek et al.^17^, Brau et al.^19^, Raayai-Ardakani et al.^20^, Jiang et al.^21^. studied the initial stage and post-buckling behavior of bilayers with infinite-thickness substrates. Suo et al.^22^, Zhou and Xia^23^ studied the wrinkling of bilayers with finite-thickness substrates , Suo et al neglected the shear stress on the film/substrate interface, and used a variable of separation method, got a very good result. Jin^24^, Ni et al.^25^ surveyed the research progress on the buckling of thin-film soft-substrate bilayers systematically and summarized the typical instability patterns of wrinkling, creasing, delaminated buckling, folding, period-double wrinkling, and ridge formation. Thin-film surface folding is still considered to be the final buckling path in the current research, and without considering the situation of a prestressed substrate, the buckling path of a typical bilayer with an infinite-thickness substrate is wrinkling–period doubling–period quadrupling–quadruple folding.

There are physical limitations in real systems, such as skin tissue attached to bone. This system is composed of bilayers of epithelial and dermal tissue, which have different dermis thicknesses. This may lead to inaccurate predictions using the infinite-thickness substrate model. In addition, the soft colloids that are widely used in chip packaging^26^ or rubber materials on soft wire surfaces will form a hardening layer by the action of oxidation corrosion^27^ and form bilayers for different substrate thicknesses composed of inner soft materials. The surface buckling for a finite-thickness substrate model is more applicable for real systems. Thus, it is important to consider the finite-thickness substrate problem of bilayers. In addition, surface folding has been considered to be the final buckling stage in recent studies^28,29^. Any further buckling behaviors after folding will be discussed in this paper.

Herein, based on an elastic half-space method and linear hypothesis, we extended the stability theory of a beam on an elastic foundation to bilayer wrinkling. Thus, the analytical solution for film buckling with a finite-thickness substrate under a smaller load can be solved in the vicinity of the wrinkling. Then, for the post-bucking behavior during large deformation, we established a finite element model of bilayers using the Neo-Hookean hyperelastic constitutive model to simulate the buckling behaviors under various modulus ratios and thickness ratios. We also coupled the self-contact analysis method to our finite element model, so that the further buckling behaviors after folding could be determined. All of the simulation examples were calculated for surface contacted slip after folding, and the final stage of the buckling path was considered to be symmetry breaking of the surface morphology of the film. We first compared our analytical solution and the finite element model results for film wrinkling to verify the accuracy of our finite element model. The change of the buckling path of the film surface with the thickness ratio was then studied by more post-buckling simulations. The results showed that the buckling path of the film surface was significantly influenced by the thickness ratio, and with the increase in the thickness ratio, five type buckling paths were found.

**Figure 1:**
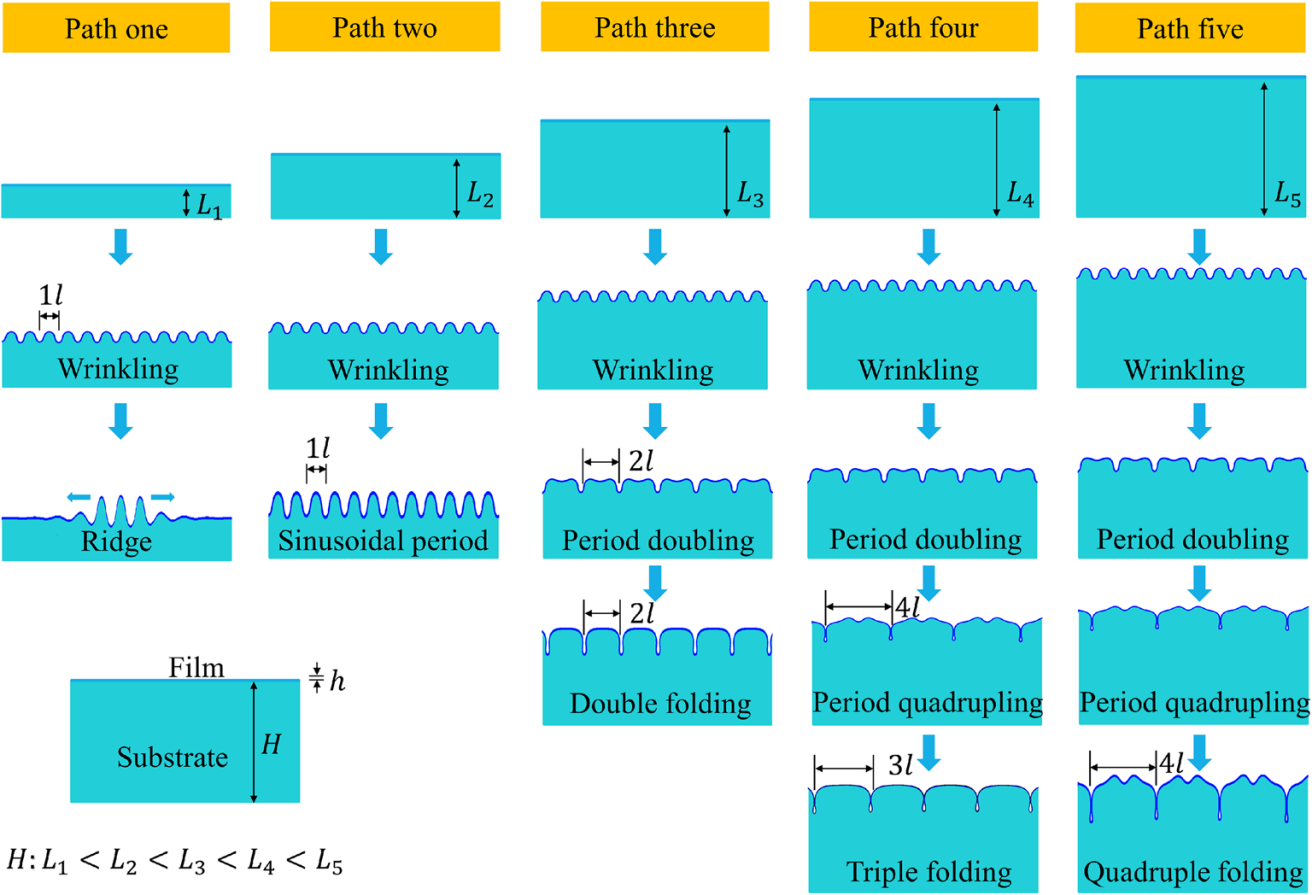
Buckling path of bilayers with different thickness ratios. Path one: wrinkling–local ridge; Path two: wrinkling–sinusoidal period; Path three: wrinkling–period doubling–double folding; Path four: wrinkling–period doubling–period quadrupling–quadruple folding–triple folding; Path five: wrinkling–period doubling–period quadrupling–quadruple folding.

The typical buckling path was path five, corresponding to an infinite-thickness substrate. This was consistent with the results of Xu^16^, and we further compared with other previously reported experimental data^15^. Path one was a local ridged buckling evolution, which was similar to the surface ridge for prestressed substrates reported previously^13,30^. However, the local ridge of path one with a very small thickness ratio and very large modulus ratio exhibited a different behavior, i.e., with increasing load, the local ridge of the film surface stopped increasing. In contrast, the adjacent wave crest began to increase and continued until all of the wave crests increased. Finally, the surface morphology became a sinusoidal periodic mode, similar to wrinkling. This mode was similar to the telephone cord buckling reported previously^31–33^, in which the variations of the buckling behaviors of annealed telephone cord films under high compressive stresses with temperature were reported. Path two also appeared when the substrate was very thin, but unlike path one, after slightly increasing the substrate thickness, the surface of the film retained a sinusoidal periodic mode after wrinkling, and new buckling modes did not appear. Path three involved a double period mode after wrinkling in the film surface, but unlike path five, with increasing load, the period quadrupling did not appear, but presented a new buckling mode, i.e., the upward wave trough of the formed double period mode disappeared, and a new morphology of the film surface that resembled a double folding appeared. In path four, the complete buckling evolution of Wrinkling–period doubling–period quadrupling–quadruple folding occurred. Upon further loading, the quadruple folding diminished due to contact slip of the film surface, and after a period of asymmetric evolution, a stable and symmetric triple folding appeared. This final buckling stage of path four was similar to that reported previously^30,34^, in which the triple folding of bilayers occurred under substrate prestressing. All of the paths are shown in Fig. 1.

Finally, the buckling path changes with different modulus ratios and thickness ratios was given in our paper.

## Wrinkling mode of bilayers in small deformation

**Figure 2:**
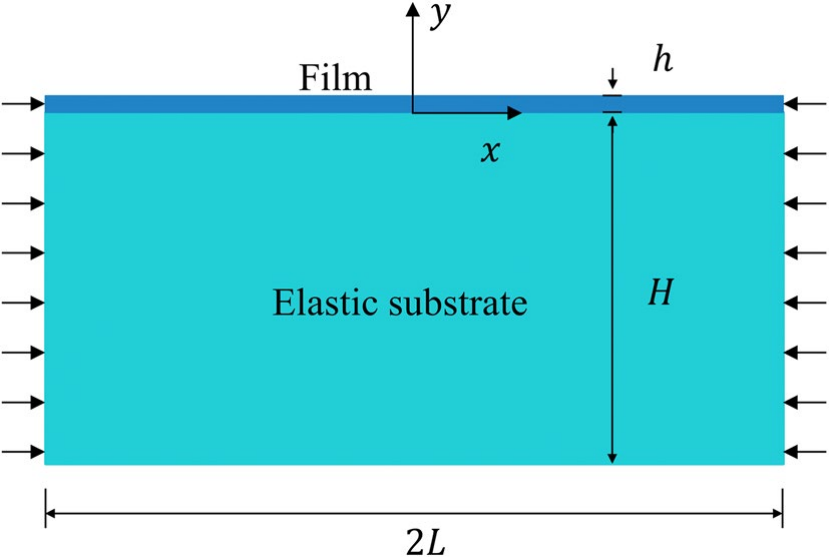
Thin-film soft-substrate bilayers.

The thin-film soft-substrate bilayers can be regarded as an incompressible film attached to an elastic half space, and we can use the traditional elastic space method to solve this problem. We assume that the film is incompressible with a length of 2*L* and a thickness of *h*, and it is attached to an elastic substrate of thickness *H*. The film and substrate do not undergo any relative sliding and separation (Fig. 2).

The deflection of thin film is denoted as *w*(*x*), and we assumed that the wrinkling of the bulk structure was periodic with period ${l_0}$ under a symmetric displacement load ${\varepsilon _n}$ at both ends. The load ${\varepsilon _n}$ satisfies the following relations:

1 $$\begin{aligned} \frac{{{\varepsilon _n}}}{L} = \frac{1}{{{l_0}}}\mathop \smallint \nolimits _0^{{l_0}} (1 - \sqrt{1 - {w_x}^2} )dx. \end{aligned}$$

The force of the elastic substrate acting on the film was assumed to be ${P_y}$ (any point ${P_y}$ along the vertical direction). We considered the differential equation of the deflection of a beam on an elastic foundation. In the case of plane strain, this differential equation is as follows:

2 $$\begin{aligned} D{w^{(4)}} + Q{w^{(2)}} + {P_y} = 0, \end{aligned}$$

where *D* and *Q* are the bending stiffness and in-plane load, respectively, for a rectangular section, $D = {{{E_f}{h^3}} \big / {12(1 - {\mu _f}^2)}}$, $Q = {{{E_f}h\varepsilon } \big / {(1 - {\mu _f}^2)}}$, the $\varepsilon$ is the in-membrane compressive strain, ${E_f}$ is the film elastic modulus, and ${\mu _f}$ is the film Poisson’s ratio.

${P_y}$ can be obtained by coordinating the deformation of the film and substrate. We consider the situation of a small deformation. According to linear elasticity theory, the displacement in two directions, *u*(*x*, *y*), *v*(*x*, *y*), of the substrate is given as follows^15^:

3 $$\begin{aligned} \begin{aligned} 2(1 - {\mu _s}){u_{xx}} + {v_{xy}} + (1 - 2{\mu _s}){u_{yy}} =0, \\ 2(1 - {\mu _s}){v_{yy}} + {u_{xy}} + (1 - 2{\mu _s}){v_{xx}} =0, \\ \end{aligned} \end{aligned}$$

where ${\mu _s}$ is the substrate Poisson ratio.

The substrate stress ${\sigma _i}(x,y) {\text { }}(i = x,y)$ satisfies the geometric and physical equations, and we obtain the following:

4 $$\begin{aligned} \begin{aligned} {\sigma _x}(x,y) = \frac{{(1 - {\mu _s}){E_s}}}{{(1 - 2{\mu _s})(1 + {\mu _s})}}({u_x} + \frac{{{\mu _s}}}{{(1 - {\mu _s})}}{v_y}), \\ {\sigma _y}(x,y) = \frac{{(1 - {\mu _s}){E_s}}}{{(1 - 2{\mu _s})(1 + {\mu _s})}}({v_y} + \frac{{{\mu _s}}}{{(1 - {\mu _s})}}{u_x}), \\ \end{aligned} \end{aligned}$$

where the ${E_s}$ is substrate elastic modulus.

At $$y = 0$$, the vertical displacement *f*(*x*) of the substrate is coordinated with the film deflection, i.e., $$ v(x,0) = f(x) = w(x)$$, and other boundary conditions can be set to $$u(x,0) = 0,{\text { }}u(x, - H) = v(x, - H) = 0$$.

To solve Eq. (3), we use the Fourier transform. The substrate displacements *u*, *v* were transformed along the *x*-axis, yielding

5 $$\begin{aligned} \begin{aligned} {\tilde{u}}(\omega ,y) = {\mathscr {F}}\left\{ {u\left( {x,y} \right) } \right\} = \int _{ - \infty }^\infty {u\left( {x,y} \right) } {\text { }}{e^{ - i\omega x}}dx, \\ {\tilde{v}}(\omega ,y) = {\mathscr {F}}\left\{ {v\left( {x,y} \right) } \right\} = \int _{ - \infty }^\infty {v\left( {x,y} \right) {\text { }}} {e^{ - i\omega x}}dx, \\ \end{aligned} \end{aligned}$$

where ${\tilde{u}}$ and ${\tilde{v}}$ are the frequency transforms of *u* and *v*, respectively, and $\omega$ is the Fourier parameter. The frequency transforms of Eq. (3) are as follows:

6 $$\begin{aligned} \begin{aligned} 2{\omega ^2}(1 - {\mu _s}){\tilde{u}} - i\omega {{{\tilde{v}}}_y} - (1 - 2{\mu _s}){{{\tilde{u}}}_{yy}} = 0, \\ 2(1 - {\mu _s}){{{\tilde{v}}}_{yy}} + i\omega {{{\tilde{u}}}_y} - {\omega ^2}(1 - 2{\mu _s}){\tilde{v}} = 0. \\ \end{aligned} \end{aligned}$$

The boundary conditions transform to ${\tilde{u}}(\omega , - H) = {\tilde{v}}(\omega , - H) = 0,{\text { }}{\tilde{u}}(\omega ,0) = 0,{\text { }}{\tilde{v}}(\omega ,0) = {\tilde{f}}(\omega )$, where

7 $$\begin{aligned} {\tilde{f}}(\omega ) = \int _{ - \infty }^\infty {f(x)} {e^{ - i\omega x}}dx. \end{aligned}$$

Equation (6) has a derivative operation that forms a decoupling equation

8 $$\begin{aligned} \begin{aligned} {{{\tilde{u}}}^{(4)}} - 2{\omega ^2}{{{\tilde{u}}}^{(2)}} + {\omega ^4}{\tilde{u}} = 0, \\ {{{\tilde{v}}}^{(4)}} - 2{\omega ^2}{{{\tilde{v}}}^{(2)}} + {\omega ^4}{\tilde{v}} = 0. \\ \end{aligned} \end{aligned}$$

Solving Eqs. (6) and (8) with the boundary conditions, a special solution of the equation is obtained:

9 $$\begin{aligned} \begin{aligned} {\tilde{u}}(\omega ,y) = \frac{{({\Phi _1}(\omega ,y){e^{\omega (2H + 3y)}} + {\Phi _2}(\omega ,y){e^{\omega (4H + 3y)}} + {\Phi _3}(\omega ,y){e^{\omega (2H + y)}} + {\Phi _2}(\omega ,y){e^{\omega y}}){\tilde{f}}(\omega )}}{{(4{H^2}{\omega ^2} + 32{\mu _s}^2 - 48{\mu _s} + 18){e^{2\omega H}} - 16{{({\mu _s} - \frac{3}{4})}^2}({e^{4\omega H}} + 1)}},\\ {\tilde{v}}(\omega ,y) = \frac{{({\Phi _4}(\omega ,y){e^{\omega (2H + 3y)}} + {\Phi _5}(\omega ,y){e^{\omega (4H + 3y)}} + {\Phi _6}(\omega ,y){e^{\omega (2H + y)}} - {\Phi _5}(\omega ,y){e^{\omega y}}){\tilde{f}}(\omega )}}{{(4{H^2}{\omega ^2} + 32{\mu _s}^2 - 48{\mu _s} + 18){e^{2\omega H}} - 16{{({\mu _s} - \frac{3}{4})}^2}({e^{4\omega H}} + 1)}}. \end{aligned} \end{aligned}$$

where

10 $$\begin{aligned} \begin{aligned} {\Phi _1}(\omega ,y)&= 2i{e^{ - 2\omega y}}\omega ( - {H^2}\omega - \omega Hy - 2{\mu _s}y + \frac{3}{2}y), \\ {\Phi _2}(\omega ,y)&= 2i{e^{ - 2\omega y}}\omega (2y({\mu _s} - \frac{3}{4})), \\ {\Phi _3}(\omega ,y)&= 2i{e^{ - 2\omega y}}\omega ({H^2}\omega + \omega Hy - 2{\mu _s}y + \frac{3}{2}y), \\ {\Phi _4}(\omega ,y)&= 2{e^{ - 2\omega y}}(8{\mu _s}^2 + ( - 12 + (4H + 2y)\omega ){\mu _s} + \frac{9}{2} + H(H + y){\omega ^2} + ( - 3H - \frac{{3y}}{2})\omega ), \\ {\Phi _5}(\omega ,y)&= 2{e^{ - 2\omega y}}( - 2({\mu _s} - \frac{3}{4})(\omega y + 4{\mu _s} - 3)), \\ {\Phi _6}(\omega ,y)&= 2{e^{ - 2\omega y}}(8{\mu _s}^2 + ( - 12 + ( - 4H - 2y)\omega ){\mu _s} + \frac{9}{2} + H(H + y){\omega ^2} + (3H + \frac{{3y}}{2})\omega ). \\ \end{aligned} \end{aligned}$$

The solution is the displacement of the finite-thickness substrate. In the general case, we can obtain an accurate analytical solution of any initial buckling mode using Eq. (9).

We next solve Eq. (2) in the Fourier space. The expression is rewritten as

11 $$\begin{aligned} {\omega ^4}D{\tilde{v}}(\omega ,0) - {\omega ^2}Q{\tilde{v}}(\omega ,0) + {\mathscr {F}}\left( {{P_y}} \right) = 0, \end{aligned}$$

where ${\mathscr {F}}\left( {{P_y}} \right) = {{\tilde{\sigma }} _y}(x,0)$ is the Fourier transform of the substrate stress function. By Eq. (4), we obtain

12 $$\begin{aligned} {{\tilde{\sigma }} _y}(x,y) = \frac{{(1 - {\mu _s}){E_s}}}{{(1 - 2{\mu _s})(1 + {\mu _s})}}({{\tilde{v}}_y} + \frac{{i\omega {\mu _s}}}{{(1 - {\mu _s})}}{\tilde{u}}). \end{aligned}$$

Using Eq. (9), the effect of the substrate ${\mathscr {F}}\left( {{P_y}} \right)$ is

13 $$\begin{aligned} {\mathscr {F}}\left( {{P_y}} \right) = \Psi (\omega ){\tilde{f}}(\omega ). \end{aligned}$$

All the effects of the substrate constitutive relations and boundary conditions are included in the function $\Psi (\omega )$, as follows:

14 $$\begin{aligned} \Psi (\omega ) = \frac{{4({\mu _s} - 1){E_s}(2{\omega ^2}H - (4{\mu _s}\omega - 3\omega )\sinh (2\omega H))}}{{({\mu _s} + 1)(4{\omega ^2}{H^2} + {{(4{\mu _s} - 3)}^2}(2 - 2\cosh (2\omega H)))}}. \end{aligned}$$

Using Eqs. (9), (11), and (13), we obtain the governing equation of the frequency space:

15 $$\begin{aligned} \left( {{\omega ^4}D - {\omega ^2}Q + \Psi (\omega )} \right) {\tilde{f}}(\omega ) = 0. \end{aligned}$$

In the case of a small deformation, a presumptive displacement function ${\tilde{f}}(\omega )$ is included, and the vicinity of the initial buckling solution can be obtained by Eq. (15). To study this problem more comprehensively, we used a function $\mathrm{Z}$ to represent Eq. (15):

16 $$\begin{aligned} \mathrm{Z}(\omega ) = \left( {{\omega ^4}D - {\omega ^2}Q + \Psi (\omega )} \right) {\tilde{f}}(\omega ) \equiv 0. \end{aligned}$$

We define an intermediate function $g(\omega )$ to determine the relationship of the buckling parameters. $g(\omega )$ is assumed to be

17 $$\begin{aligned} g(\omega ) = 4{\omega ^2}{H^2} + {(4{\mu _s} - 3)^2}(2 - 2\cosh (2\omega H)). \end{aligned}$$

Using the Eq.(16) and Eq.(17), combined with integral operation, easy to get

18 $$\begin{aligned} \int _{ - \infty }^\infty {\mathrm{Z}(\omega )g(\omega )} d\omega = 0. \end{aligned}$$

The integral relation in Equation (18) can be simplified, and the following compressive strain is obtained:

19 $$\begin{aligned} \varepsilon = \frac{{\int _{ - \infty }^\infty {{\omega ^4}D{\tilde{f}}(\omega )g(\omega )d} \omega + \int _{ - \infty }^\infty {\Psi (\omega ){\tilde{f}}(\omega )g(\omega )d} \omega }}{{\int _{ - \infty }^\infty {{\omega ^2}\overset{\frown }{Q} {\tilde{f}}(\omega )g(\omega )d} \omega }}, \end{aligned}$$

where $\overset{\frown }{Q} = {{{E_f}h} \big / {(1 - {\mu _f}^2)}}$. The right side of Eq. (19) will no longer be related to parameter $\omega$ after the integral operation. We now analyze the wrinkling mode of the film. We assumed the Poisson’s ratios ${\mu _f}$ and ${\mu _s}$ were both 0.433^15^ to simulate a soft material. Meanwhile, we were more interested in the relationship between the buckling mode and the thickness ratio. Thus, the modulus ratio was assumed to 600. We selected the displacement functions $f(x) = a\cos (kx)$ and ${\tilde{f}}(\omega ) = \pi (\delta (\omega + k) + \delta (\omega - k))$, we set the critical compression strain ${\varepsilon _0}$ of the initial buckling stage to an extreme value of $\varepsilon$, and the initial wave number set to ${k_0}$.

**Figure 3:**
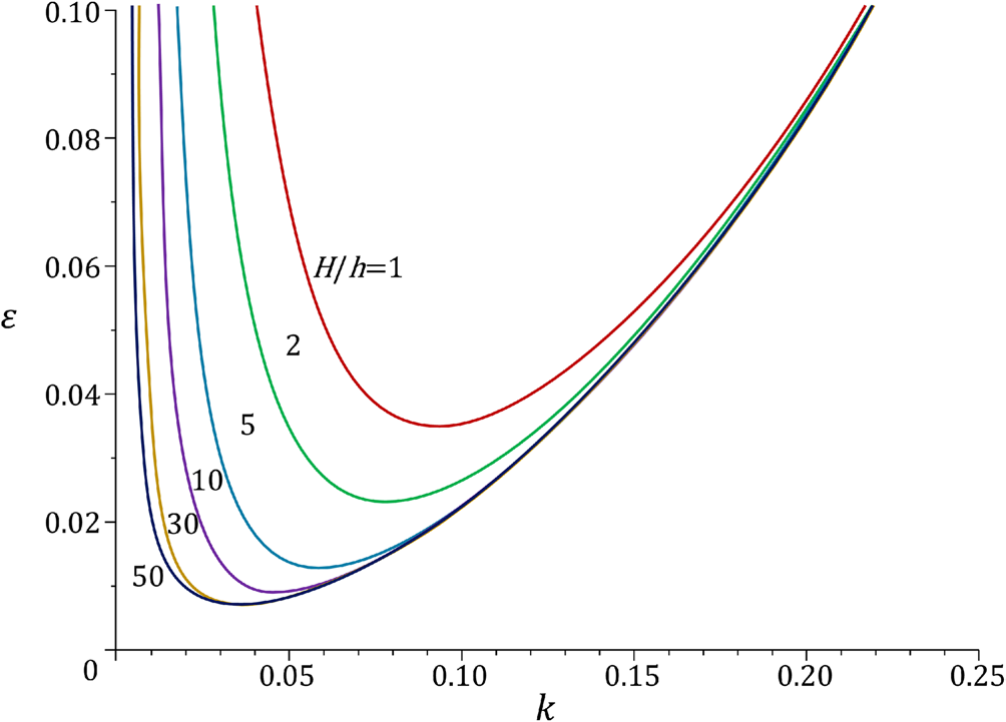
Relationship between the compression strain and the wavenumber given by Eq. (19). The modulus ratio was assumed to be 600, and ${\mu _f}$ and ${\mu _s}$ were assumed to be 0.433. The different curves represent the different thickness ratios.

This coincided with the experimental observation that the film surface morphology retained a sinusoidal period during surface wrinkling. Further results of Eq. (19) for different thickness ratios are given in Fig. 3, which shows that the compression strain of wrinkling changed with the thickness ratio. The case with ${H \big / h} = 1$ often occurs when the substrate is attached to a harder object, such as bones or wires^26,27^. Meanwhile, with the increase in ${H \big / h}$, the extreme point of the curve moved downward such that the critical wavenumber and strain were reduced. When ${H \big / h} = 50$, further increases in ${H \big / h}$ produced negligible changes of the curves, so the substrate could be regarded as an infinite-thickness substrate. The extreme point of the curve provides the critical compression strain. In the general case, the thinner the substrate is, the smaller the critical wave number becomes, and the amplitude from Eq. (1) is larger.

## Finite element model of thin-film soft-substrate bilayers

The initial buckling stage of the thin-film soft-substrate bilayers appeared as a small deformation. Although we have given an analytical solution, the post-buckling of large deformation is still difficult to solve. Brau et al.^15^ analyzed the infinite substrate buckling behavior used the Green strain. A governing equation of large displacement was provided, the PDEs were solved by a perturbation method and verified with experimental data, and an approximate method to predict the film buckling of wrinkling and period doubling was presented. However, due to the high nonlinearity of the problem, this method cannot incorporate the hyperelastic constitutive relation of a soft material. We consider the post-buckling of a finite substrate. It is difficult to obtain a mathematical solution, but the finite element method (FEM) provides good approximations^16,23,35^.

Based on the powerful FEM, to obtain a more profound understanding of the physical process of film deformation, we considered the contact-sliding of a film after the film was folding. This process used the augmented Lagrangian method based on a penalty function^36,37^ that is widely used in finite element analysis of adhesive friction contact, sliding friction contact, and frictionless contact problems. To ensure the correctness of the numerical simulation, a hexahedral mesh was selected, the element size of the film was set to 0.4*h* along *x*-axis and 0.2*h* along the *y*-axis. For the substrate, the element size was same as film thickness along the *x*-axis, and the element size of the *y*-direction was gradually increased from 0.2 h to 8 h. The film and substrate were compatibly deformed.

**Figure 4:**
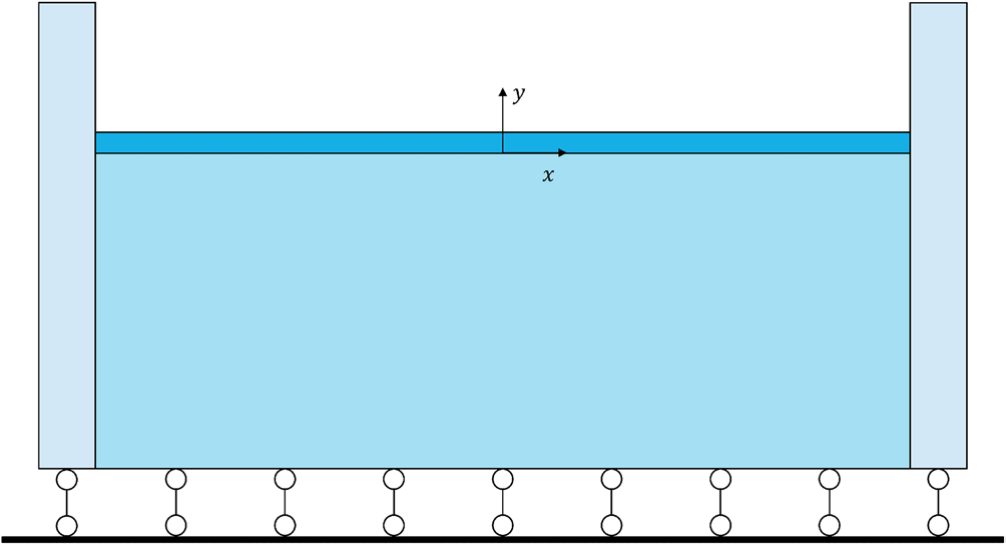
Schematic diagram of finite element analysis model of thin-film soft-substrate bilayers.

The two-sided displacement load of the finite element model was applied using the rigid columns shown in Fig. 4. The rigid column and model were set with a contact element with zero friction (to speed up convergence, the frictional force of the film and the rigid column can be set to smaller values initially and then set to zero after wrinkling, but it is not mandatory). We considered the contact slip of the film surface, a self-contact element was set on top of film. The *y*-direction displacement of the element model was limited at the bottom. Meanwhile a displacement load was applied to the rigid columns. Otherwise, the large deformation of the buckling was considered. We used the Solid 185 element of the ANSYS software and the Neo-Hookeam hyperelastic constitutive model. All the simulations were completed using the ANSYS software, and part of the theoretical calculations were aided by the MAPLE software. We adopted the default convergence criterion of ANSYS to ensure the reliability of the calculation results.

### Initial stage buckling of analytical solution and FEM

**Figure 5:**
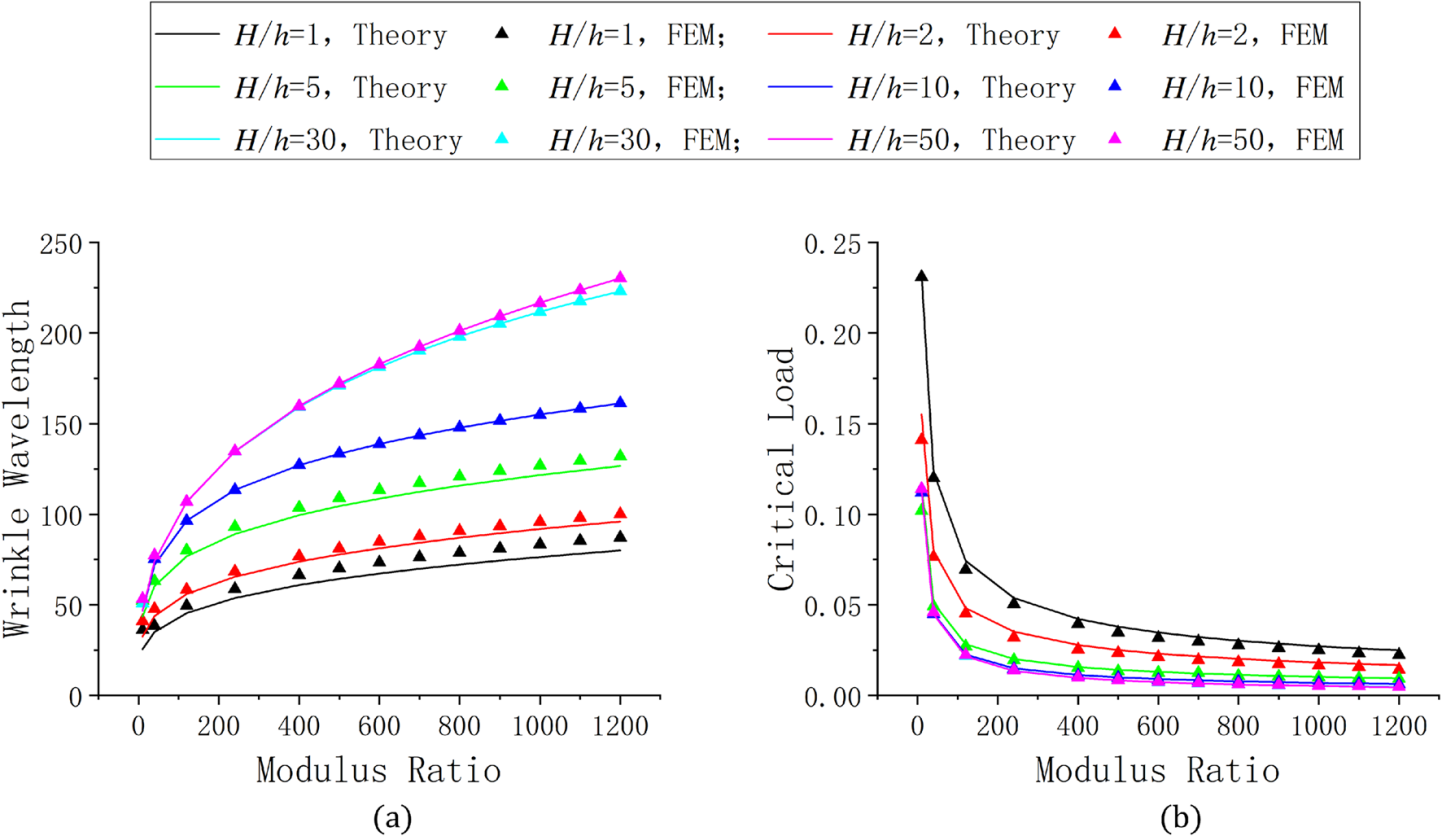
Result comparison of the results of the finite element method (FEM) and Eq. (19). The different curves show the effect of the thickness ratio: (**a**) FEM and theoretical solutions of wrinkling wavelength change with modulus ratio, and (**b**) FEM and theoretical solutions of critical load.

**Figure 6:**
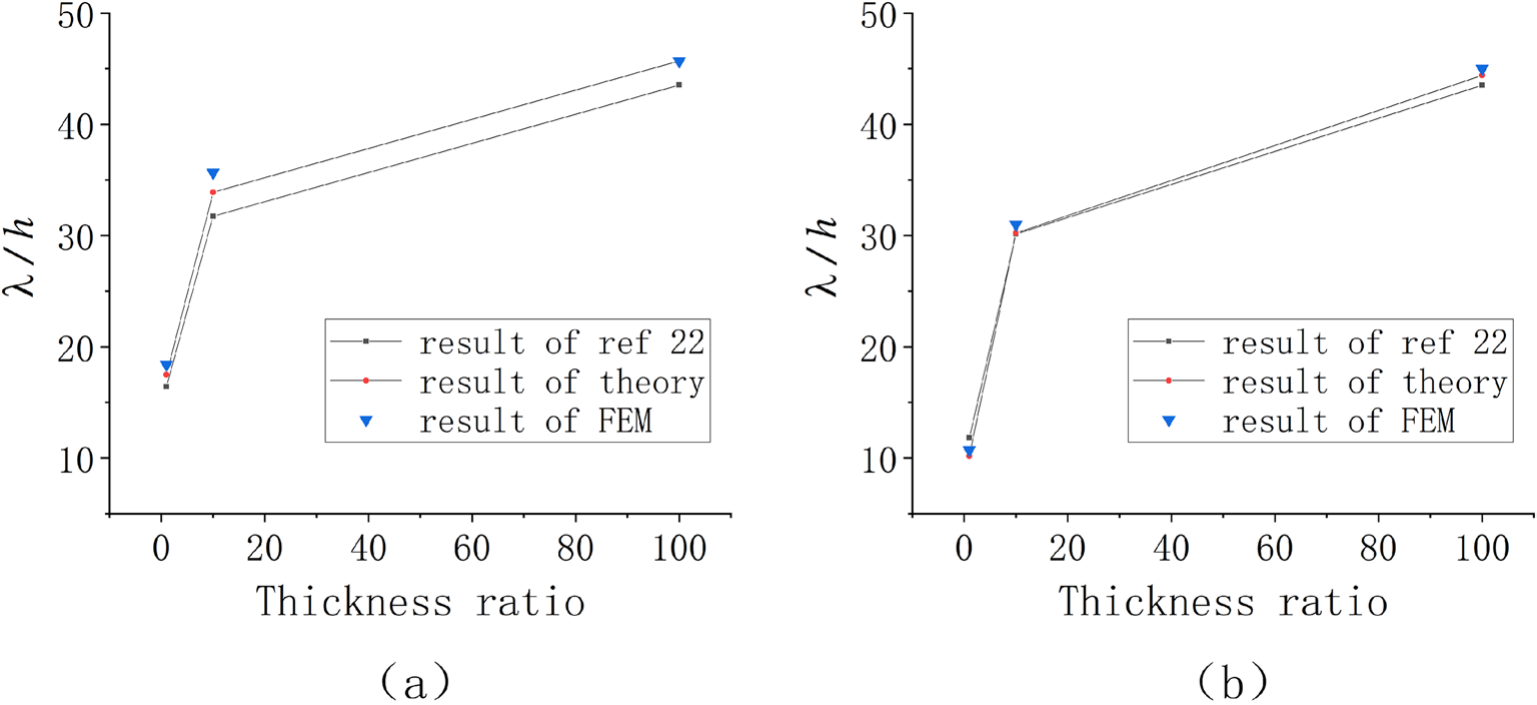
Comparison of the results of ref 22 with the theoretical solution and the FEM results: (**a**) The modulus ratio is 1000 , the poisson’s ratio of substrate is 0.4, the poisson’s ratio of film is 0.3 (**b**) poisson’s ratio of substrate is 0.5, the other parameters are the same as in (**a**).

We investigated the wrinkling of the finite element model under small loads. In this case, the Neo-Hookean hyperelastic constitutive model was similar to a linear elastic constitutive model, allowing us to compare our finite element model with the analytical solution proposed in Section 2. We considered different modulus ratios and thickness ratios, critical wavelengths ${\lambda _0}$, and critical loads ${\varepsilon _{n0}}$ of wrinkling obtained by the finite element model and Eq. (19), as shown in Fig. 5. When the thickness ratio was large, the FEM and theoretical solutions of the wrinkling wavelength agreed closely. However, when the substrate thickness was reduced, our FEM solution was larger than that of Eq. (19), which may possibly because of that there is a competition between sinusoidal mode and local ridge mode. In the case of thin substrate, the influence of local ridge mode is amplified synchronously. So, with the comprehensive influence, although the film is still wrinkled in a sinusoidal mode, there are still systematic differences of the wavelength between the idealized theoretical solution and the FEM simulation. For the case of a thick substrate, the FEM simulation is in good agreement with the theoretical results. The calculations also show that the FEM and theoretical solutions of the critical load agreed closely (as shown in Fig. 5b). We also compared the results of ref 22 with the theoretical solution and the FEM results we proposed, as shown in Fig. 6. Through the results, the solutions obtained with different boundary conditions are slightly different, and the FEM solution is a little higher than the theoretical solution.

### Buckling path of local ridge

Due to physical constraint of the substrate in a bilayer system, the inital buckling mode shows a sinusoidal period mode. The film surface morphology also generally exhibited a sinusoidal period after wrinkling. However, when the modulus ratio was very large and the thickness ratio was very small, our simulation showed a different buckling evolution in which the sinusoidal period mode became a local ridged mode. With increasing load, the adjacent wave crests began to increase until all of the wave crests increased, and the surface of the film exhibited a sinusoidal period mode again.

**Figure 7:**
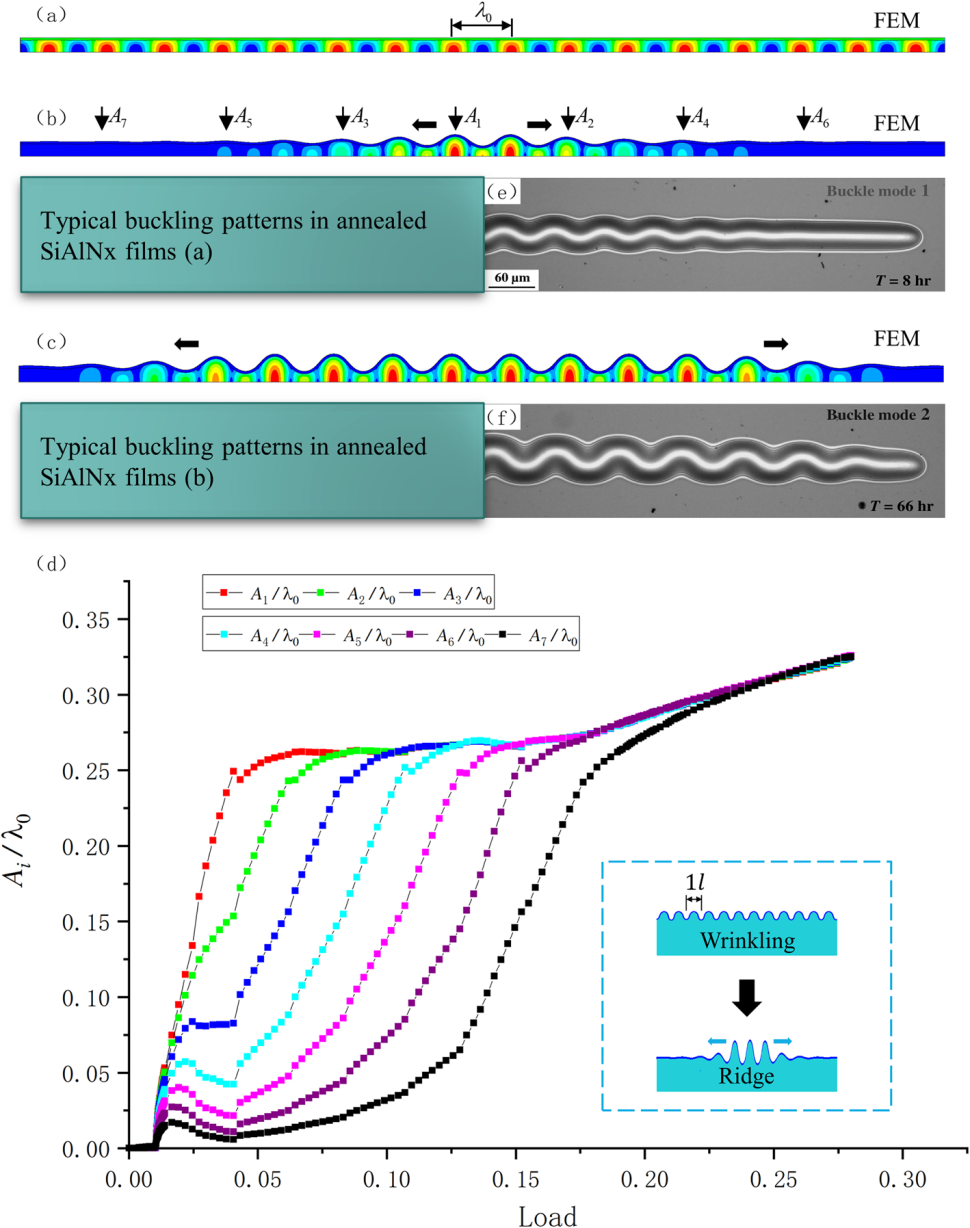
FEM solution of thin-film soft-substrate bilayers with a modulus ratio of 600 and a thickness ratio of 5: (**a**)–(**c**) FEM simulation results of the bilayer buckling path; (**d**) Observed amplitude changes with the increase in load (the color contours represent the different wave crest) ; (**e**), (**f**) The experimental result reported by^32^.

Based on the comparison of the FEM solution and previously reported results^32^, for the conditions in our FEM simulations, the elastic contribution of the substrate can be neglected due to the very large modulus ratio and very small thickness ratio. However, the bottom edge restriction cannot be ignored. Thus, the buckling path of the bilayers in this case is similar to the buckling of a film in contact with an air layer and fixed on a stiff substrate. Our FEM simulation results were also similar to the experimental results reported by^32^. In their experiment, the confined air layer enclosed in the thin layer can be assumed as an elastic substrate with very small stiffness (the elastic stiffness here is very small, and the influence of shear stiffness is ignored). Accordingly, the antisymmetric axial plane of the warped thin layer is similar to the fixed lower surface of our finite element model. So, the thin layer and the air layer of the experiment can form a bilayers with small thickness ratio and large modulus ratio as shown in Fig. 7. The FEM solution of thin-film soft-substrate bilayers with a modulus ratio of 600 and a thickness ratio of 5 was analyzed. The buckling path was wrinkling, followed by local ridge formation, and finally, sinusoidal period structure formation. As shown in Fig. 7b and c, our FEM simulation results were similar to previously reported experimental results^32^. We selected a symmetric model and observed the amplitude change of seven wave crests (for dimensionless purposes, ${A_i}/{\lambda _0}$ was chosen), and all of the solutions were entered and shown in Fig. 7d (the several lines prior to bifurcation in the figure are overlapping, and amplitudes are following the same paths).

### Buckling path of bilayers with large thickness ratio

**Figure 8:**
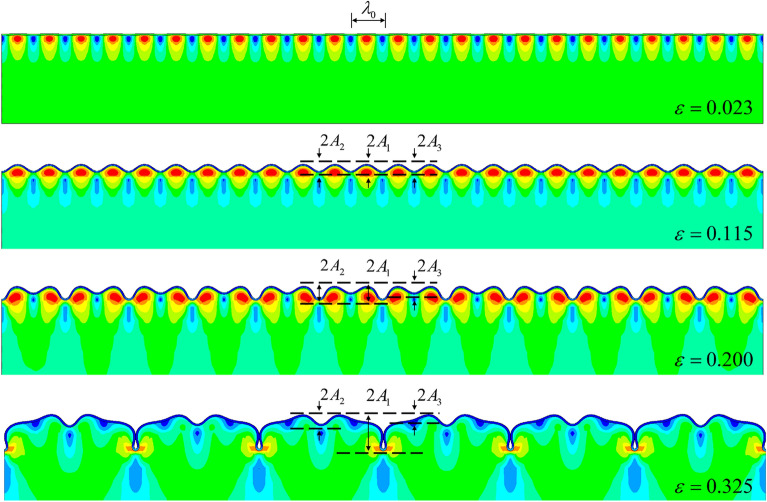
FEM solution of thin-film soft-substrate bilayers with a modulus ratio of 120 and a thickness ratio of 60. The buckling sequence of the film was wrinkling, followed by period doubling, and finally, quadruple folding.

We next analyzed the post-buckling of thin-film soft-substrate bilayers. To compare with experimental data^15^, we set the modulus ratio to 120, and the thickness ratio was 60. The simulation results are shown in Fig. 8.

**Figure 9:**
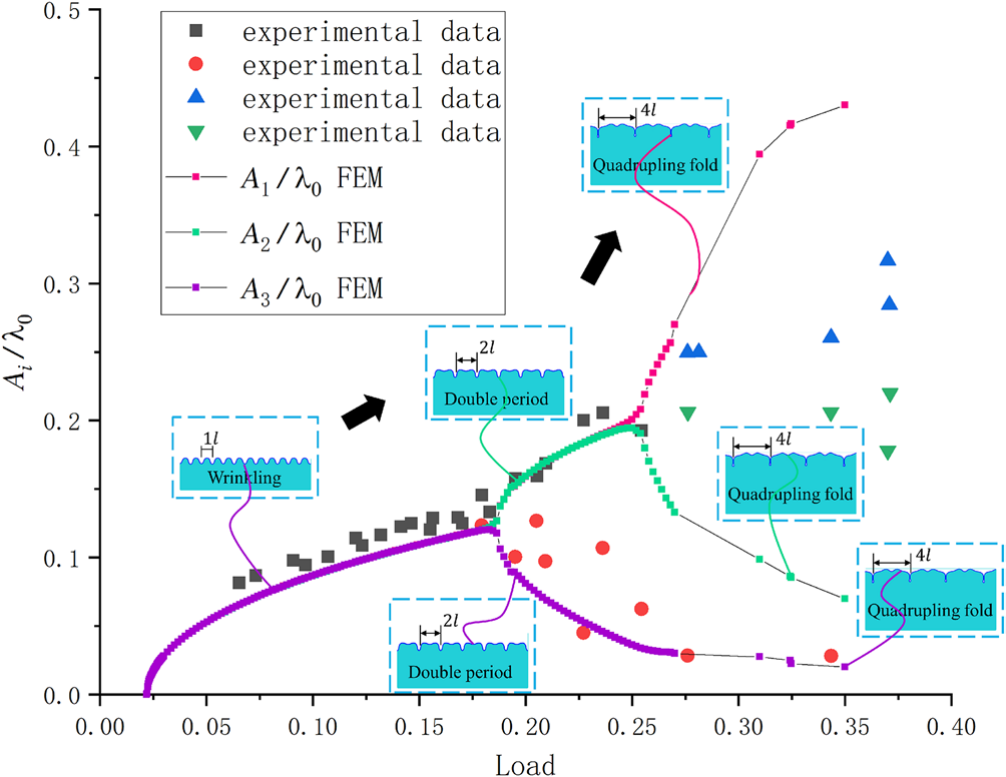
Comparison of the FEM results and experimental data^15^ of the observed amplitude changes with the increase in load. The curves correspond to our FEM simulations (the color contours represent the different wave crest), and the points are experimental data from Reference^15^.

The observed amplitude changes of ${A_1}$, ${A_2}$, and ${A_3}$ are shown in Fig. 9 as the load was increased (the several lines prior to bifurcation in the figure are overlapping, and amplitudes are following the same paths). The comparison shows that our FEM method provides a suitable method for predicting the wrinkling–period doubling mode. As for the quadruple period, there were some errors between the FEM solution and the experimental data. However, the simulation results using a lattice model method showed the same error trend as that of previously reported data^16^.

The buckling path shown in Fig. 8 is the typical evolution of the thin film with a very large substrate thickness. To distinguish this from other paths, it is denoted as path five. In this path, the final buckling stage was a quadruple folding of the film surface. A further increased load could not form any new symmetric periods but broke the quadrupling mode, causing the surface morphology to be disordered.

**Figure 10:**
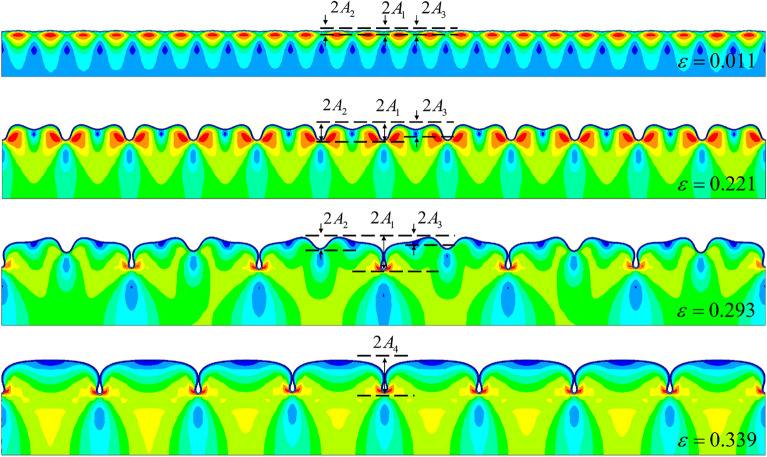
FEM simulation of path four. In this case, the quadruple folding was not the final buckling stage, but a triple folding occurred when considering the contact slip of the film surface. The observed amplitude changes are shown in Figure 11.

**Figure 11:**
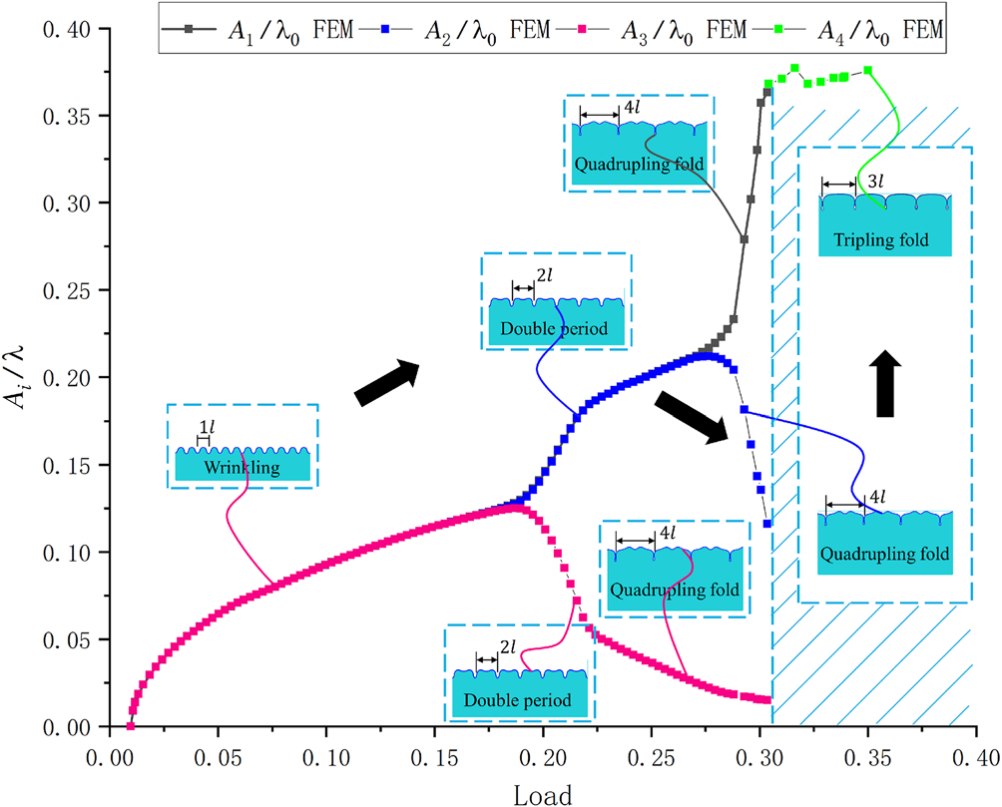
Amplitude changes of ${A_1}$, ${A_2}$, ${A_3}$, and ${A_4}$ with the increase in the load (the color contours represent the different wave crest). ${A_1}$, ${A_2}$, and ${A_3}$ are the typical buckling modes(the several lines prior to bifurcation in the figure are overlapping, and amplitudes are following the same paths). However, the change of ${A_4}$ corresponded to quadruple symmetry breaking and triple symmetry formation.

We next reduced the thickness ratio, and our FEM simulation gave the path four mode shown in Fig. 10. In this example, the thickness ratio was set to 45, and the modulus ratio was set to 400. The result of our simulation revealed a new morphological transformation, i.e., the quadruple folding to the triple folding (Fig. 11).

**Figure 12:**
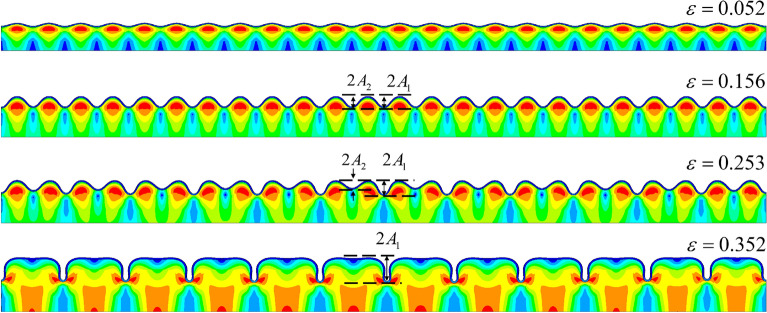
FEM simulation of path three for a modulus ratio of 240 and thickness ratio of 20. The film surface buckling mode was wrinkling–period doubling–double folding, and unlike paths four and five, due to the constraint of the substrate, the amplitude of ${A_3}$ did not appear.

Paths two and three are formed by further reducing the substrate thickness. To simulate these situations, we selected example thickness ratios of 20 and 10 with a modulus ratio of 240. Figure 12 shows path three for a thickness ratio of 20. The upward wave trough after the period doubling disappeared but formed a double folding mode.

**Figure 13:**
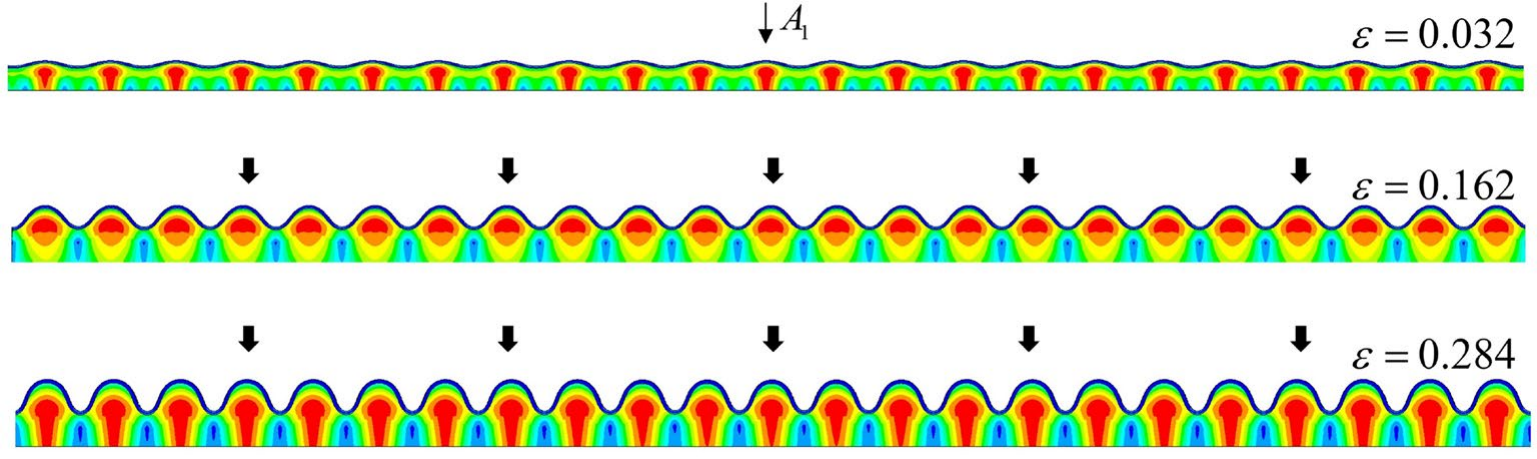
FEM simulation with a modulus ratio of 240 and thickness ratio of 10. Here, the buckling mode only had a sinusoidal period, and we observed the amplitude change of ${A_1}$.

**Figure 14:**
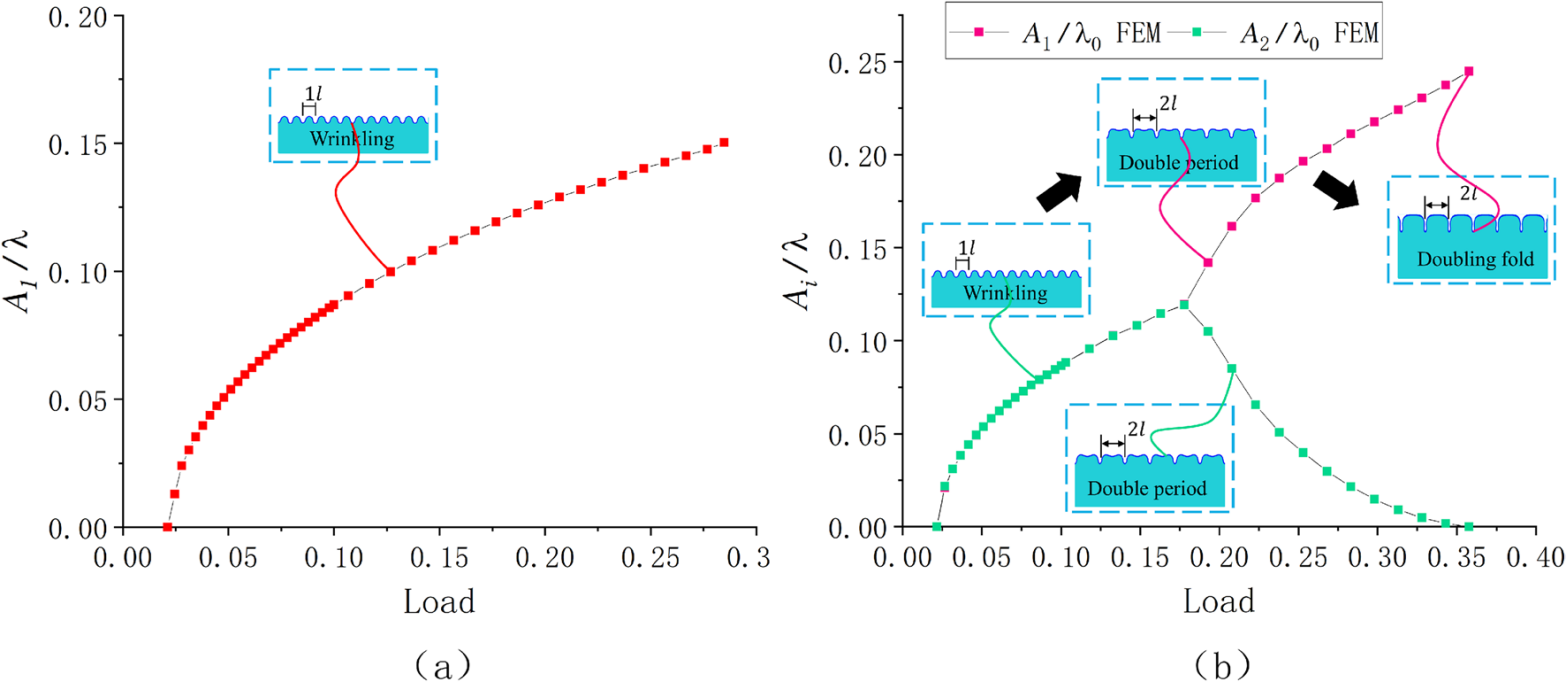
Amplitude changes of paths two and three: (**a**) observed changes of path two, and (**b**): observed changes of path three (the color contours represent the different wave crest).

We are most interested in the amplitude changes of ${A_1}$ and ${A_2}$ in Fig. 12. Similarly, Fig. 13 shows the FEM simulation of path two, in which the film retained a sinusoidal period structure after wrinkling . The amplitude changes of paths two and three are shown in Fig. 14a and b (the several lines prior to bifurcation in the figure are overlapping, and amplitudes are following the same paths), respectively.

### Results and analysis

**Figure 15:**
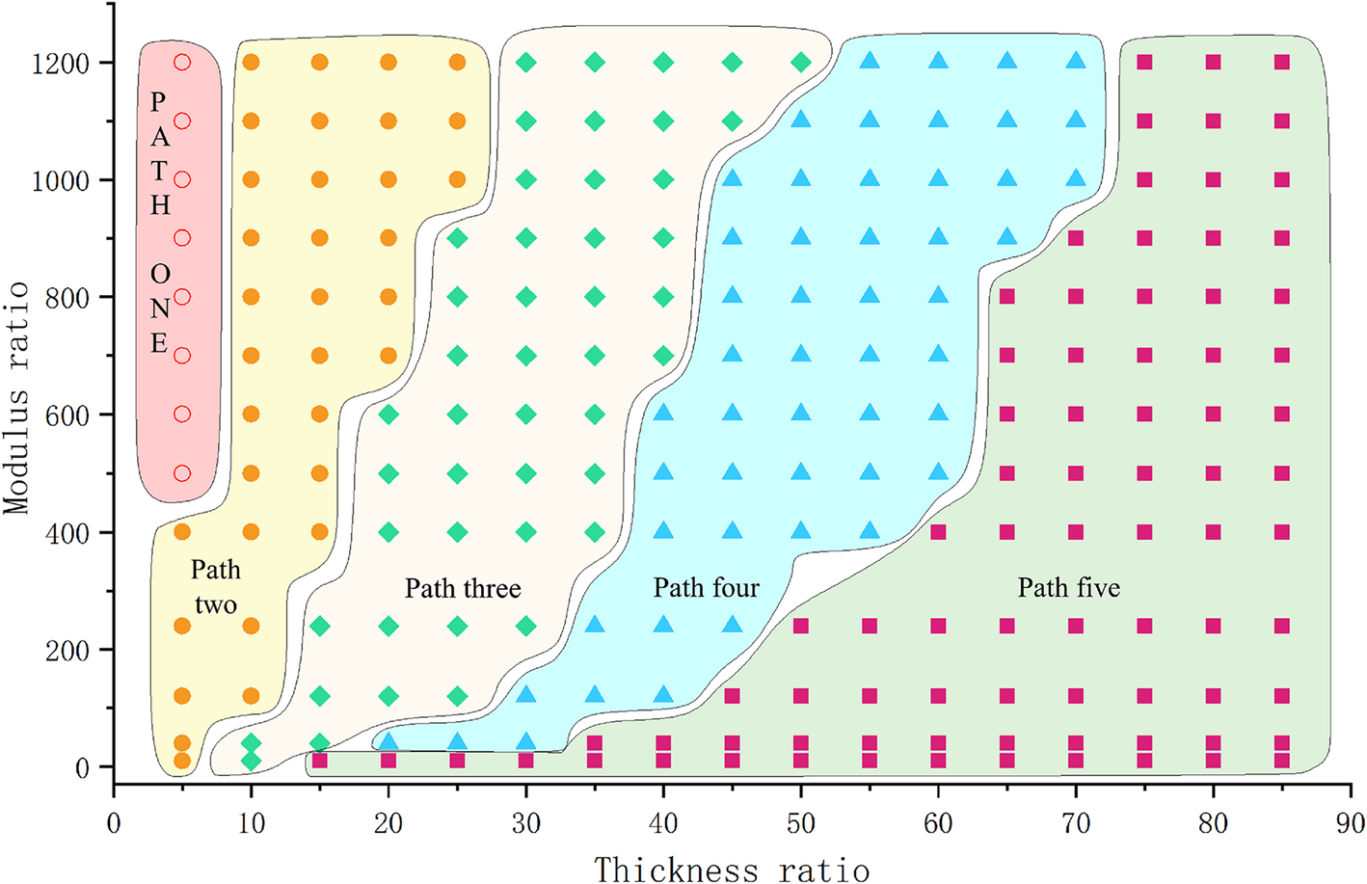
FEM simulation results for different modulus ratios and thickness ratios. In this two-dimensional image, every point corresponded to a simulation. The different areas belong to the different buckling paths. The buckling path sequence from left to right is from one to five.

We analyzed the FEM simulations with different modulus ratios and thickness ratios, and all of the results are presented as the two-dimensional image shown in Fig. 15. Paths one to five show the buckling evolution of the thin-film soft-substrate bilayers, and different modulus ratios and thickness ratios produced different buckling paths.

## Concluding remarks

This paper considers the actual physical limitations of thin-film soft-substrate bilayers with the finite-thickness substrates. The buckling evolution path simulations of the bilayers were simulated by the FEM method with different modulus ratios and thickness ratios with compression loads imposed on both sides. We also extended the buckling theoretical analysis to bilayers with finite thicknesses. The general solution of an arbitrary surface deflection with a linear elasticity model was given to verify the correctness of the FEM method. The post-buckling behavior of large deformation was simulated by our FEM model.

Our calculations showed that the film buckling went through five paths with the change of the thickness ratio of the film and substrate. We hope our results will play a role in predicting or adjusting the film surface morphology after buckling.

Finally, there is some error between our FEM results and the experimental data in the final buckling stage, which may be related to the selection of our constitutive relationship. Perhaps a new constitutive equation such as consider of the strain stiffening model of Gent or Ogden may be contribute to reduce the error of simulations.

## References

[1] Li B, Cao Y-P, Feng X-Q, Gao H (2012). Mechanics of morphological instabilities and surface wrinkling in soft materials: A review. Soft Matter..

[2] Genzer J, Groenewold J (2006). Soft matter with hard skin: From skin wrinkles to templating and material characterization. Soft Matter..

[3] Wang Z, Servio P, Rey A (2021). Biaxial nanowrinkling in cholesteric surfaces: Egg carton surfaces through chiral anchoring. Colloid Interface Sci. Commun..

[4] Rofouie P, Pasini D, Rey AD (2015). Nano-scale surface wrinkling in chiral liquid crystals and plant-based plywoods. Soft Matter..

[5] Sharon E, Roman B, Swinney HL (2007). Geometrically driven wrinkling observed in free plastic sheets and leaves. Phys. Rev. E.

[6] Cerda E, Mahadevan L (2003). Geometry and physics of wrinkling. Phys. Rev. Lett..

[7] Budday S, Steinmann P, Kuhl E (2015). Secondary instabilities modulate cortical complexity in the mammalian brain. Philos. Mag..

[8] Budday S, Steinmann P, Kuhl E (2014). The role of mechanics during brain development. J. Mech. Phys. Solids.

[9] Sun B (2018). Capillary wrinkling scaling laws of floating elastic thin film with a liquid drop. Sci. China-Phys. Mech. Astron.

[10] Zhou, R. Application of cement stabilized crushed stone construction technology in road base construction. *Modern Manuf. Technol. Equip.* (2017).

[11] Wong W, Guo T, Zhang Y, Cheng L (2010). Surface instability maps for soft materials. Soft Matter..

[12] Huang Z, Hong W, Suo Z (2004). Evolution of wrinkles in hard films on soft substrates. Phys. Rev. E.

[13] Cao, Y. & Hutchinson, J. W. Wrinkling phenomena in neo-hookean film/substrate bilayers. *J. Appl. Mech.* **79** (2012).

[14] Cao Y, Hutchinson JW (2012). From wrinkles to creases in elastomers: The instability and imperfection-sensitivity of wrinkling. Proc. R. Soc. A Math. Phys. Eng. Sci..

[15] Brau F (2011). Multiple-length-scale elastic instability mimics parametric resonance of nonlinear oscillators. Nat. Phys..

[16] Cheng Z, Xu F (2021). Intricate evolutions of multiple-period post-buckling patterns in bilayers. Sci. China Phys. Mech. Astron..

[17] Pocivavsek L (2008). Stress and fold localization in thin elastic membranes. Science.

[18] Jin, L., Auguste, A., Hayward, R. C. & Suo, Z. Bifurcation diagrams for the formation of wrinkles or creases in soft bilayers. *J. Appl. Mech.* **82** (2015).

[19] Brau F, Damman P, Diamant H, Witten TA (2013). Wrinkle to fold transition: influence of the substrate response. Soft Matter..

[20] Raayai-Ardakani S, Luis Yagüe J, Gleason KK, Boyce MC (2016). Mechanics of graded wrinkling. J. Appl. Mech..

[21] Jiang H (2008). Finite width effect of thin-films buckling on compliant substrate: Experimental and theoretical studies. J. Mech. Phys. Solids.

[22] Huang Z, Hong W, Suo Z (2005). Nonlinear analyses of wrinkles in a film bonded to a compliant substrate. J. Mech. Phys. Solids.

[23] Zhou, H. Y. & Xia, Q. The finite element simulation of surface wrinkling of rigid films on a compliant thin-substrate. In *Applied Mechanics and Materials*, vol. 446, 22–26 (Trans Tech Publ, 2014).

[24] Jin, L. *Mechanical Instabilities of Soft Materials: Creases, Wrinkles, Folds, and Ridges*. Ph.D. thesis, Harvard University (2014).

[25] Ni Y, Liu P, Ma L, Li S, He L (2018). Nonlinear buckling mechanics of film-substrate systems: Recent progress. Chin. J. Solid Mech..

[26] Suryanarayana D, Hsiao R, Gall TP, McCreary JM (1991). Enhancement of flip-chip fatigue life by encapsulation. IEEE Trans. Comp. Hybrids Manuf. Technol..

[27] Seng-shan, W. *et al.* The effect of the composition of ldpe/mvq hybrid rubber on its thermal ageing properties. *Electric Wire Cable* **2** (2007).

[28] Sultan, E. & Boudaoud, A. The buckling of a swollen thin gel layer bound to a compliant substrate. *J. Appl. Mech.* **75** (2008).

[29] Sun J-Y, Xia S, Moon M-W, Oh KH, Kim K-S (2012). Folding wrinkles of a thin stiff layer on a soft substrate. Proc. R. Soc. A Math. Phys. Eng. Sci..

[30] Auguste A, Jin L, Suo Z, Hayward RC (2014). The role of substrate pre-stretch in post-wrinkling bifurcations. Soft Matter..

[31] Thouless M (1993). Combined buckling and cracking of films. J. Am. Ceramic Soc..

[32] Yu S-J (2014). Morphological selections and dynamical evolutions of buckling patterns in sialnx films: from straight-sided to telephone cord or bubble structures. Acta Materialia.

[33] Faou J-Y, Parry G, Grachev S, Barthel E (2012). How does adhesion induce the formation of telephone cord buckles?. Phys. Rev. Lett..

[34] Budday S, Kuhl E, Hutchinson JW (2015). Period-doubling and period-tripling in growing bilayered systems. Philos. Mag..

[35] Nikravesh S, Ryu D, Shen Y-L (2020). instabilities of thin films on a compliant substrate: Direct numerical simulations from surface wrinkling to global buckling. Sci. Rep..

[36] Simo JC, Laursen T (1992). An augmented lagrangian treatment of contact problems involving friction. Comput. Struct..

[37] Stefancu AI, Melenciuc SC, Budescu M (2011). Penalty based algorithms for frictional contact problems.. Buletinul Institutului Politehnic din lasi. Sectia Constructii Arhitectura.

